# Reconstruction of Traumatic Defect of the Lower Third of the Leg Using a Combined Therapy: Negative Pressure Wound Therapy, Acellular Dermal Matrix, and Skin Graft

**DOI:** 10.1155/2014/783812

**Published:** 2014-08-11

**Authors:** Sergio Brongo, Domenico Pagliara, Nicola Campitiello, Corrado Rubino

**Affiliations:** ^1^Department of Medicine and Surgery, Plastic Surgery Unit, University of Salerno, Azienda Ospedaliera Universitaria OO.RR. San Giovanni di Dio e Ruggi d'Aragona, 1 Via San Leonardo, 84131 Salerno, Italy; ^2^Department of Orthopedic, Traumatologic, Rehabilitative and Plastic-Reconstructive Sciences, Second University of Naples, 3 L. De Crecchio, 80138 Naples, Italy

## Abstract

The reconstruction of lower third of the leg is one of the most challenging problems for plastic and reconstructive surgeons and current approaches are still disappointing. We show an easy option to obtain a coverage of traumatic pretibial defects with good aesthetic and functional results: the association of negative pressure wound therapy, acellular dermal matrix, and skin graft. The choice of this combined therapy avoids other surgical procedures such as local perforator flaps and free flaps that require more operating time, special equipment, and adequate training.

## 1. Introduction

The reconstruction of traumatic soft tissue defects in the distal third of the leg is one of the most challenging problems in lower limb surgery. Among the most widely used techniques direct closure, skin grafting, local flaps, and free flap are worthy of note. Usually, the low mobility of the surrounding skin does not make a direct closure possible. However, wound edge approximation shows a high percentage of failure or requires long time to achieve complete healing [1]. Skin graft compared to wound edge juxtaposition shows an advantage in success rate and in healing time [1]. However, for major defects skin graft does not provide optimal coverage of the underlying structures (vessels, nerves, and tendons). Even the coverage with flaps shows some disadvantages. A random flap has an indistinct perfusion pattern which requires a careful assessment of length-to-width ratio to ensure viability. These features make random flaps difficult to perform in the lower leg and anyway associated with a high rate of necrosis [2]. Musculocutaneous flaps are widespread in leg reconstruction for their reliability. However, these flaps have few indications in the distal third of leg due to the impossibility to reach the site of injury [3]. Local fasciocutaneous flaps can be harvested without a careful assessment of length-to-width ratio but still show a considerable necrosis rate in the lower third of the leg [4]. Local perforator flaps and free flaps are good options in reconstruction of traumatic defects of lower third of the leg. Local perforator flaps have a similar perfusion to musculocutaneous flaps but save the underlying fascia and muscles, resulting in less postoperative morbidity of donor site. In this type of flaps, the main risk is related to vascular complications associated to pedicle torsion and deformation. To reduce this risk, it is necessary to identify perforator with at least 1 mm in diameter [5]. Before or during surgery, the handheld doppler probe and the color doppler are reliable techniques to determine the size of the perforator [6, 7]. As free flaps, local perforator flaps require a microsurgical procedure but the microanastomoses are not needed. Therefore, these flaps have a shorter operating time compared to free flaps. Free flaps require more operating time, special equipment, and adequate training. In addition, Melissinos and Parks [8] reported that success rate of free flap was only 95.6% in reconstruction of defects of lower extremities (versus 96.8%, 100%, and 98.8% of head and neck, trunk, and upper extremities reconstruction, resp.). Keeping in mind all the previous limitations of each technique, we described a simple technique for lower third of the leg reconstruction. We used the association of negative pressure wound therapy (NPWT), acellular dermal matrix (ADM), and skin graft in a patient with traumatic defect of the lower third of the leg.

## 2. Presentation of Case

**Figure 1:**
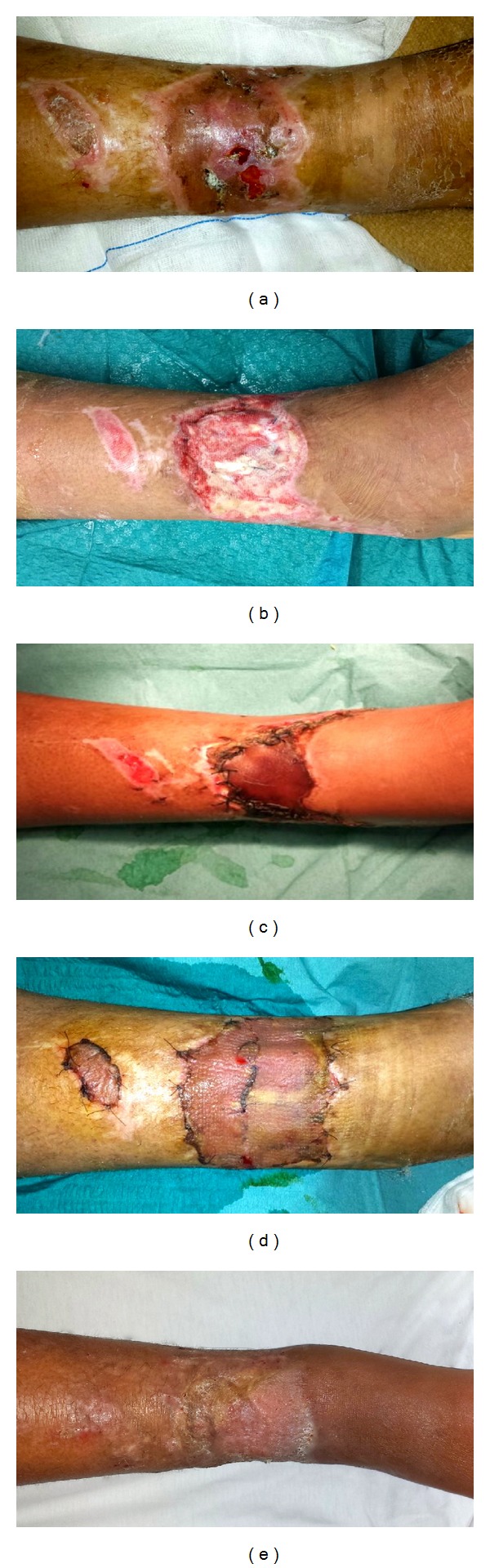
(a) Preoperative aspect before surgical debridement. (b) After debridement, the exposure of extensor hallucis longus and digitorum longus muscles tendons can be observed. (c) Aspect of wound covered using an acellular dermal matrix (ADM). (d) A partial thickness skin graft placed on regenerated dermal tissue. After debridement, ADM placement, and skin grafting we used a negative pressure wound therapy (NPWT). (e) Optimal aesthetic outcome was achieved at long-term follow-up (6 months after skin grafting).

An 18-year-old woman was involved in a high speed road traffic collision. According to the emergency services, the patient was ejected from the vehicle and landed on the road. After checking the stability of the vital parameters, the patient underwent orthopedic treatment for bilateral femur fracture. Therefore, she came to our attention with an extensive soft tissue necrosis on the lower third of the right leg (8 cm long, 15 cm wide). A thorough surgical debridement was performed. Following debridement, the wound site showed exposure of the tendons of the extensor hallucis longus and digitorum longus muscles. Therefore, the wound was covered using NPWT for four weeks, setting the system at an 80 mmHg alternating cycle. Every 5 days, wound dressings were replaced and the negative pressure was applied again. Sequential wound assessment demonstrated the growth of granulation tissue. At the end of the 4th week of NPWT, the wound was covered using ADM in order to obtain an optimal coverage of tendons. After surgical debridement of granulation tissue in excess, the ADM was modeled according to the shape of defect and was fixed to the surrounding tissues. Following the ADM placement, NPWT was reapplied over the ADM. The NPWT system was set at a lower pressure (50 mmHg alternating cycle) for 14 days, changing the wound dressings every 5 days. In this way, we anticipated to 14 days (rather than 21) the removal of the silicone layer that covers the porous matrix of ADM. At the 14th day after the placement of ADM the dermal tissue was completely regenerated and showed the typical golden-yellow color. A split-thickness skin graft harvested from the anterior face of left thigh was used to cover the ADM. We reapplied the NPWT on the graft at a 50 mmHg negative pressure, in order to keep it clean and accelerate the engraftment. After a week of NPWT, complete integration of the graft was achieved. The coverage of the underlying tendons was optimal (Figure 1) and functional outcome was satisfying with a good tendons sliding in the flexion-extension movements of the toes.

## 3. Discussion

The reconstructive options for lower third of the leg are still few and disappointing. We combined NPWT, ADM, and skin graft, leading to a good aesthetic result with an optimal tendons coverage and normal sliding in flexion and extension movements of the toes. A similar treatment was documented by a retrospective review of the Walter Reed Army Medical Center on 16 patients with blast wounds and exposure of tendons and bones [9]. In 15 of 16 patients a definitive coverage was obtained, with the need to repeat the ADM placement only in two cases. We used the NPWT three times: after the initial debridement to keep the wound clean, after placement of ADM to prevent the adherences formation with the underlying tendons, and after grafting to speed the engraftment. NPWT improves the success rate of split-thickness skin grafts and reduces the rate of repeated grafting [10]. This effect is linked to several factors [11–14]: the uniform contact between the graft and the wound bed, the removal of wound fluid with prevention of hematoma or seroma formation under the graft, the reduced risk of infection, and the moist environment due to the occlusive nature of wound dressing that prevents the drying of the graft. NPWT may exercise the same positive effects after placement of ADM, with documented cellular microdeformations that would lead to an increased fibroblasts proliferation and dermal regeneration [15, 16].

As underlined by Graham et al. [17] in a recent retrospective study on ten patients with traumatic degloving injuries, the placement of ADM and following split-thickness skin graft are a good therapeutic option when tendons and bones are exposed. As is well known, the exposure of tendons and bones compromises the vascularity of the wound bed required for engraftment. In soft tissue injuries with exposure of tendons and absence of paratenon, the main risk is tendons adherences formation that would result in decreased range of motion. However, a recent prospective study [18] showed a normal range of motion after reconstruction with ADM in 32 patients with soft tissue defects of the upper or lower extremity. So ADM provides an adequate safety profile for functional restoration in patients with soft tissue defects and exposure of tendons.

## 4. Conclusion

The association of NPWT, ADM, and skin graft provides an easy option to obtain a lasting coverage of traumatic defects on lower third of the leg, with good aesthetic and functional results.

The therapeutic process is not time consuming; a portable device to perform the NPWT at home is available. The portable device is for single use with a lifespan of up to 7 days. Therefore, in outpatient settings we can remove the dressing every 5 days and replace the portable NPWT device. The choice of NPWT-ADM-skin graft combined therapy avoids other surgical procedures such as musculocutaneous flaps, local perforator flaps, and free flaps in patients who reject the associated scars and donor site morbidities.
